# Robust averaging of emotional faces and its association with psychotic-like experiences and social connection

**DOI:** 10.1038/s41598-026-35374-z

**Published:** 2026-01-10

**Authors:** Katie Gibbs, Xiaoyu Dong, Yunsu Shin, Steven M. Silverstein, David Dodell-Feder

**Affiliations:** 1 Department of Psychology, University of Rochester, 500 Joseph C. Wilson Blvd, Rochester, NY 14627 USA; 2 Department of Psychological and Brain Sciences, Washington University in St. Louis, St. Louis, USA; 3 Department of Psychiatry, University of Rochester Medical Center, Rochester, USA; 4 Department of Neuroscience, University of Rochester Medical Center, Rochester, USA; 5 Department of Ophthalmology, University of Rochester Medical Center, Rochester, USA

**Keywords:** Robust averaging, Social cognition, Social connection, Psychosis, Psychotic-like experiences, Neuroscience, Psychology, Psychology

## Abstract

Robust averaging is an analytic feature of our perceptual systems that adaptively downweights outlying information during information processing. Here, we test whether individuals demonstrate robust averaging for a critical source of social information—facial affect—whether it is altered by psychotic-like experiences, and whether it is associated with social connection (the positive sense of relatedness from relationships and perceived/received support and inclusion). Participants completed a novel face averaging task in which they judged whether face arrays that varied as a function of reliability (variance of the faces), strength (emotional intensity of the faces), and valence (positive or negative), were on average more positive or negative. Afterwards, participants completed self-report measures of psychotic-like experiences and social connection. Two analytic approaches revealed the presence of robust averaging for emotional faces whereby inlying faces (i.e., those closer to the mean emotion expression of the face array) were given greater weight compared to outlying faces on trial-by-trial decisions. This effect was specific to high variance trials. There were no associations between robust averaging and social connection or psychotic-like experiences. These findings suggest individuals use robust averaging as an adaptive strategy to summarize social information, although any clinical and behavioral implications of individual differences remain to be clarified.

**Supplementary Information:**

The online version contains supplementary material available at 10.1038/s41598-026-35374-z.

## Introduction

The sensory information we encounter in the world is inherently noisy. The job of perception is to integrate this information and extract a meaningful signal while adaptively ignoring noise. As a way of accomplishing this task, research suggests that the visual system represents sets of similar items using summary statistics through a process known as ensemble coding^1,2^. This process leads to gist-like perception, where the characteristics of a large group can be discerned rapidly^3^, allowing the visual system to process stimuli without delays due to attention^4–6^ and working memory^7^ limitations. However, the mechanisms underlying ensemble perception are still largely unclear^3,5,6^.

One line of work regarding ensemble perceptions has aimed to clarify *how* we minimize noise from sensory signals when generating summary representations. Research suggests that when we encounter conflicting perceptual information, we evaluate the strength and reliability of the stimuli to integrate the information for decision-making^8,9^. This is analogous to decision-making in statistics, where one considers the strength (i.e., mean) and reliability (i.e., variance) of empirical evidence. Specifically, during decision-making, individuals adaptively assign less weight to extreme or outlying sensory information in a process known as “robust averaging,” similar to downweighting, rather than excluding, statistical outliers^8,9^. This process, which may have parallels to a focus on global versus local modes of processing^10,11^, is beneficial, because over-weighting extreme or outlying observations can lead to faulty judgment and decision making. Of course, there are also situations where attending to outliers is essential, such as in certain types of visual search (i.e., identifying a suspicious person) or novelty detection (i.e., making note of an unusual observation). When atypical or salient stimuli carry important information, making judgements based on the group average may be maladaptive. Thus, in everyday life, weighting of outlying information likely depends on relevance and goals. Here, we focus on situations where it is more important to extract and focus on the mean of an array rather than outlying information; that is, situations that would benefit from robust averaging. Previous research has established that robust averaging occurs for low-level stimuli such as color^8,9^, but it is unclear if it occurs for higher-order ensemble representations, such as social information.

Of all the types of perception we perform, the perception of social information is perhaps most challenging. Social information is fundamentally “fuzzy”^12^ and ambiguous^13^, requiring inferences about largely or partially unobservable internal states. And yet, our ability to resolve this fuzziness and ambiguity may carry important social consequences, especially because ensemble representations of social information can provide information about crowds, environments, and social interactions that can only be conveyed at a group-level^14^. For example, perceiving the overall threat of a crowd, such as whether expressions suggest a group intends to harm you versus help you, and the direction the group is heading cannot be conveyed by individual faces alone^15–17^. Although ensemble coding—the idea that the visual system represents groups of similar items using summary statistics^1,18^—has been demonstrated to occur for faces and emotional outliers^1^, and work has established that robust averaging occurs across manipulations of mean and variance for color^8,9^, it remains unclear if robust averaging best characterizes how evidence integration occurs for critical sources of social information like facial affect.

Regarding facial affect perception, research has demonstrated that information about faces can be rapidly extracted, even within 100 ms or less after stimulus onset^19–21^. Researchers have also shown that observers’ ratings of ensemble information are highly correlated with the mathematical means of perceptual items, even when observers cannot recall individual stimuli in the crowd^21^, suggesting that ensemble information underlies implicit perception. While previous studies have also shown that individuals can quickly and accurately extract the mean emotion from multiple faces with mixed valences^3,19,20^ to form ensemble representations^2,22^, there is mixed evidence regarding the influence of variance on averaging performance. Some studies suggest that individuals tend to discount outliers or use subsampling strategies when averaging faces^1^, while others report that greater variance or heterogeneity in a set impairs averaging accuracy^23,24^. Interactions between mean and variance have also been reported, with findings showing that mean perception can be moderated by variance and vice versa^5,25^. If robust averaging was used during social perception, the impact of factors like set mean and variance remains to be clarified.

Additionally, the clinical implications of robust averaging ability are unknown. It is possible that altered robust averaging may be a useful way to understand pathophysiological changes associated with certain psychological disorders, such as psychotic disorders. Many of the symptoms of psychosis can be characterized by fixed decisions or inferences about environmental stimuli (e.g., delusions, hallucinations) based on insufficient or unsupported information. In fact, information processing in psychosis is impaired to such a degree that researchers have characterized individuals with psychosis as “bad statisticians,” liberally accepting weak or noisy evidence as valid due to a lowered decision threshold^26^. In addition, schizophrenia is frequently characterized by both deficient top-down and bottom-up processing in the organization of perceptual information, resulting in impairments in integrating contextual information and creating higher-order representation of visual stimuli^27^. This could reflect attributing more weight or salience to more extreme or outlying information; in other words, reduced robust averaging.

One way to test hypotheses about psychosis-related alterations in perception and cognition is by evaluating their covariance with psychotic-like experiences (PLE). PLE are subclinical perceptions, thoughts, or odd, unusual, or delusion-like beliefs that can range significantly in form, severity, and persistence. PLEs are relatively common in the general population in the absence of a psychotic disorder^28–30^. The etiological and phenomenological similarity between PLE and psychotic disorders^31,32^, and the observation that PLE increases risk for psychotic disorders^33^, means that PLE can be thought of as an expression of one’s underlying vulnerability for a psychotic disorder. As such, we would expect to see individuals with PLE exhibit reduced ability to make use of adaptive perceptual strategies, such as robust averaging. Since social cognitive and functioning disturbances are often observed in individuals experiencing PLE and psychotic disorders^34,35^, it is possible that reduced robust averaging of social information may be a contributing mechanism.

In support of some of these ideas, Larsen et al.^8^ found that robust averaging of low-level color perception is less likely to occur in individuals experiencing PLE. The authors used a perceptual averaging task to prompt participants to make judgements about the average color (red or blue) of a stimulus array with varying strength (i.e., mean color of the array) and reliability (i.e., variance of the items in the array). They found that hallucination-prone individuals appeared to weigh inlying and outlying evidence more equally, demonstrating impairments in evidence integration and robust averaging in psychosis-prone individuals.

Lastly, if robust averaging did occur during social perception, it would be useful to determine its association with real world social connection. While ensemble perception has been shown to be affected by emotional states such as anxiety^36^ and mood^37^, which could in turn impact social relationships, little is known about the extent to which robust averaging of social information impacts relationships. Social connection is a composite of the structural (e.g., network size, diversity), functional (e.g., social support), and qualitative aspects (e.g., perceived connection, satisfaction) of social relationships^38,39^ that has been identified as critical for health and well-being^38–40^. Given that robust averaging may facilitate making judgements about unfamiliar social partners, groups, and environments, it is possible that difficulties with this process may relate to suspiciousness, impaired social connection, or decreased quality of relationships that is typical of individuals with psychotic experiences and disorders.

In consideration of these issues, the current study aims to evaluate the presence of robust averaging in social perception and its association with PLE and social connection. We tested our aims in a non-clinical sample since our primary goal was to evaluate the presence of robust averaging during typical social perception. While robust averaging deficits have not yet been demonstrated in a clinical sample, robust averaging deficits of low-level stimuli (e.g., color) have been previously demonstrated in a non-clinical, psychosis-prone group^8^. As such, the current study builds on this finding and seeks to extend the prior findings to higher-order (i.e., social) processing in a non-clinical population. The benefit of examining robust averaging in a non-clinical sample is that the influence of PLE can be examined without the confounds associated with psychotic illness (e.g., medication effects, executive functioning and memory impairments). Further, as described, PLE are relatively common in the general population^31,32^ making it possible to study psychosis-spectrum-related variance in a non-clinical sample. And, given the phenomenological, etiological, and pathophysiological continuity between psychotic-like experiences in the general population and psychotic disorders^32,32^, findings of altered social robust averaging as a function of PLE here would very likely converge with those from a clinical sample. Although this would need to be confirmed in a separate study, evaluating how robust averaging varies as a function of PLE is a useful first step.

To evaluate these aims, we had participants perform a novel facial affect averaging task based on existing robust averaging paradigms^8,9^, and self-report PLE and aspects of social connection (e.g., social support). We used the facial averaging task data to determine the extent to which participants downweighted faces that were outlying in terms of facial affect valence intensity (e.g., an extreme, negatively valenced face in a largely positive valenced group of faces). We predicted that individuals adaptively downweight the influence of outliers in perception when making decisions related to facial affect, indicated by inlying faces (i.e., faces with valence intensities lying closer to the mean) having a larger impact on trial-by-trial decisions than outlying faces. Additionally, we predicted that increased robust averaging will occur with greater variability in stimuli, in line with previous findings^8^. We also expect that individuals who experience more PLE will show a decrease in robust averaging, indicated by a smaller difference between the impact of inlying and outlying faces on decision-making. Lastly, we predicted that robust averaging would be associated with adaptive social behavior, and thus individuals who exhibit increased robust averaging will also have higher scores on social connection measures (e.g., decreased loneliness).

## Methods

### Transparency and openness

The current study was preregistered on the Open Science Framework (https://osf.io/wmnbg). De-identified data and analysis code from this study are available on the Open Science Framework at the following link: https://osf.io/w596j/. All data were analyzed using R Statistical Software^41^ (v.4.4.3) and R Studio using the lavaan^42^ (Version 0.6-19), semTools^43^ (Version 0.5-7), lme4^44^ (Version 1.1-36), psych^45^ (Version 2.5.3), rstatix^46^ (Version 0.7.2), WRS2^47^ (Version 1.1-6), and effectsize^48^ (Version 1.0.0) packages.

### Participants

207 participants were recruited through the University of Rochester Department of Psychology’s study pool (SONA) during the Fall 2024-Spring 2025 academic year. Enrollment was open to individuals of any sex, gender, race, and ethnicity who were at least 18 years old, fluent in English, and had normal or corrected-to-normal vision. One participant was excluded from analysis due to not completing the task, resulting in a final sample of 206. Participants were on average 20 years old (*SD* = 1.3, range = 18–25), predominately female at-birth (74%), self-identified as female (71%; 25% male, 4% non-binary or other), racially Asian (41%; 33.5% White; 11% Black or African American, 0.5% American Indian or Alaska Native; 8% Multiracial; 6% other or prefer not to answer) and non-Hispanic/Latino (88%; 9% Hispanic/Latino, 3% prefer not to answer; Table 1). All participants provided written informed consent, including consent for broad data sharing on data repositories, and were compensated for their time by receiving partial course credit. This study was approved by the University of Rochester Research Subjects Review Board (RSRB). All aspects of the study were performed in accordance with RSRB guidelines and regulations.

**Table 1:** Participant demographics.

	Mean	SD	Range	Clinical cutoff score	No (%)	Yes (%)
Age (years)	20.3	1.3	18–25			
PLE						
RGPTS-R	9.2	7.1	0–30	16^a^	163 (79%)	43 (21%)
RGPTS-P	4.7	6.7	0–36	11^a^	177 (87%)	27 (13%)
PDI	4.5	3.1	0–14	8^b^	170 (83%)	36 (17%)
CAPS	2.3	2.6	0–12	–	–	–
Social connection						
Loneliness	36.9	10.0	20–67			
MSPSS	66.0	13.0	34–84			
FNSS	53.5	12.6	17–70			
	*n* (%)					
Sex						
Female	153 (74%)					
Male	53 (26%)					
Gender						
Female	147 (71%)					
Male	52 (25%)					
None of the above	5 (2%)					
Enby	2 (1%)					
Race						
Asian	85 (41%)					
White	69 (34%)					
Black or African American	22 (11%)					
American Indian or Alaska Native	1 (0.5%)					
Multiracial	16 (8%)					
Other/Prefer not to answer	13 (6%)					
Ethnicity						
Non-Hispanic/Latino	181 (88%)					
Hispanic/Latino	19 (9%)					
Prefer not to answer	6 (3%)					

^a^Clinical significance/cut-off scores for the RGPTS-R and RGPTS-P scales come from Freeman et al.^49^.

^b^Clinical significance/cut-off scores for the PDI come from Preti et al.^50^.

### Sample size determination and power

Based on the effects observed by Larsen et al.^8^ who used a similar task and design with non-social stimuli, we aimed to recruit at least 162 participants, which would provide 80% power to detect the expected effects using the analytic strategy described below (alpha = .05, two-tailed). We set a more conservative target *N* = 200 to account for the possibility that the effects in Larsen et al. were overestimates and because our paradigm differed from Larsen et al. in several respects (e.g., social stimuli, fewer trials). Our final sample size of *N* = 206 provided > 89% power to detect expected effects.

### Robust averaging task

**Fig. 1:**
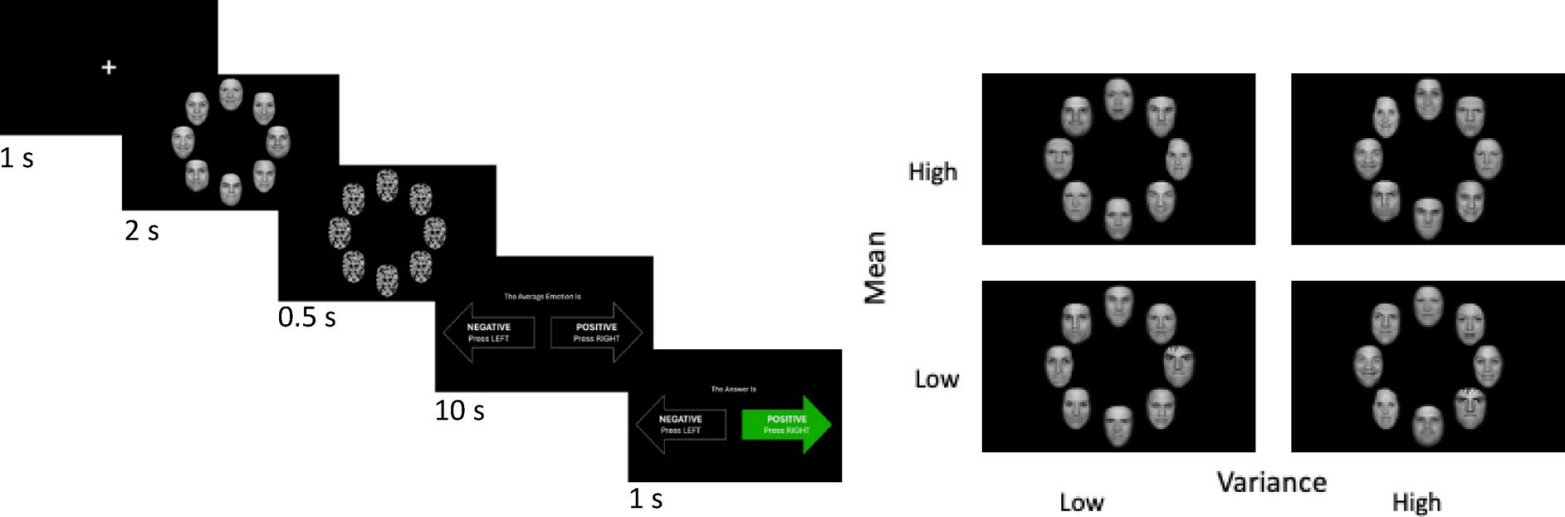
Facial averaging paradigm. (Left) Depiction of a single trial of the facial averaging paradigm along with timing. Each trial began with 1 s of fixation on a central cross, followed by 2 s of an 8-face array, .5 s mask, and 10 s for participants to make a response, after which they received feedback on their performance. Faces are from the NimStim Set of Facial Expressions, which consists of posed photographs of professional actors who provided their informed consent for the use of their images in research. (Right) Depiction of sample stimuli for each trial type across high/low levels of mean (using a median split) and variance.

All participants completed a robust averaging task (Fig. 1) based on work from prior groups^8^. Participants were presented with an 8-face element stimulus array presented in a circle around a central cross with faces varying in average emotion intensity between very negative and very positive. Facial stimuli were from Ji and Pourtois^51^ and included 16 identities of professional actors differing in sex, race, and ethnicity and ranging across angry, happy, and neutral expressions that came from the validated NimStim Set of Facial Expressions^52^. Face images were morphed between angry (Face 1) and happy (Face 50) expressions for all eight female and male identities (morphed stimuli from Ji and Pourtois are available on the Open Science Framework, 10.17605/OSF.IO/UFJMK). Face arrays were generated at the start of each experiment for each participant whereby faces were drawn randomly to have a specific mean, sampled from a Gaussian distribution centered on the midpoint face value of 25 (neutral face), and variance, which we defined as low with a *SD* = 5 or high with a *SD* = 15. As face values were being randomly sampled from a predefined set of faces values (1–50), there were small deviations in the actual array mean and *SD* from the predefined ones. Means had to be within 5% of the predefined value (the one sampled from the Gaussian distribution) or else the array was regenerated, up to 500 times. The percentage of trials in which the array mean differed from the predefined one by more than 5% was minimal (.8% of all trials). The degree of trial-wise deviation in *SD* was also small, with the actual *SD* being close to the target value of either 5 or 15 (*SD* value of low variance trials: *M* = 5.34, *SD* = .15; *SD* value of high variance trials:* M* = 15.88, *SD* = .77). These specific *SD* values were determined based on prior work using the current stimulus set^51^ and pilot testing, and were similar in relative magnitude to those of Larsen et al.^8^ (i.e., high variance trials being 3 times the amount of low variance trials). Stimuli were randomized per participant, where at the start of the task, PsychoPy sampled face intensities according to the design. Consequently, the experimental design was the same across participants, but the specific face combinations differed. Participants first completed 10 trials of a practice task. Subsequently, participants completed 500 trials of the main experimental task (250 low variance and 250 high variance trials). Each stimulus array was presented for 2000 ms, followed by a 500 ms mask, after which participants were asked to indicate if the faces were on average “more positive” or “more negative” by pressing the left or right arrow key (mapping of emotion to key was counterbalanced). Prior studies indicate that observers can accurately and reliably extract and identify mean emotional intensity from short exposures (250–500 ms)^1,3,6^ and utilize robust averaging with low-level stimuli tasks of the same duration as ours^5,8,9^. To maximize the similarity between our task and Larsen et al., visual feedback was provided after each response to indicate whether the response was correct or incorrect. Average task completion time was 49 min (*SD* = 7). Pilot and experimental participants did not report any significant issues with task-related fatigue.

### Psychotic-like experiences measures

#### Revised green paranoid thoughts scale

Paranoia was measured using the Revised Green Paranoid Thoughts Scale (RGPTS)^53^. This scale is an 18-item self-report measure of referential (e.g., “I often heard people referring to me”) and persecutory ideation (e.g., “I was convinced there was a conspiracy against me”). Participants responded to each item using a 0 (*not at all*) to 4 (*totally*) scale. Total scores were calculated as the sum of all items. The scale demonstrated good reliability for both the reference scale (ω_*u*_ = .88) and persecution scale (ω_*u*_ = .91).

#### Peters delusion inventory

Delusion-proneness was measured using the Peters Delusion Inventory (PDI)^54^. This scale is a 21-item self-report measure of delusional beliefs (e.g., “Do you ever feel as if things in magazines or on TV were written especially for you?”). Participants rated each item *yes/no*, with *yes* responses having additional response prompts to indicate distress (rated 1–5; 1 = *not at all*, 5 = *very*), preoccupation (rated 1–5; 1 = *hardly ever*, 5 = all the time), and conviction (rated 1–5; 1 = *do not believe it is true*, 5 = *believe it is absolutely true*) in the belief. We analyzed the total number of endorsed items. The scale demonstrated good reliability (ω_*u*_ = .75).

#### Cardiff anomalous perceptions scale

Hallucination-proneness was measured using the Cardiff Anomalous Perceptions Scale (CAPS)^55^. This scale is a 32-item self-report measure of aberrant perceptual experiences (e.g., “Do you ever see shapes, lights, or colors even though there is nothing really there?”). Participants were asked to rate each item *yes/no*, with *yes* responses having additional response prompts to indicate distress (rated 1–5; 1 = *not at all*, 5 = *very*), distraction (rated 1–5; 1 = *not at all*, 5 = *completely intrusive*), and frequency (rated 1–5; 1 = *hardly at all*, 5 = *all the time*) of the experience. We analyzed the total score, which was calculated as the number of endorsed items. The scale showed good reliability (ω_*u*_ = .82).

### Social connection measures

#### UCLA loneliness questionnaire

Loneliness was measured using the Revised UCLA Loneliness Questionnaire (ULS)^56^. This scale is a 20-item self-report measure of disconnection (e.g., “I feel in tune with the people around me”, “I lack companionship”). Participants were asked to indicate how often they feel the way described in each of the item statements and respond to each item using a 1 (*never*) to 4 (*often*) scale. Total scores were calculated as the sum of all items. The scale showed good reliability (ω_*u*_ = .92).

#### Multidimensional scale of perceived social support

Social support was measured using the Multidimensional Scale of Perceived Social Support (MSPSS)^57^. This scale is a 12-item self-report measure of individuals’ perceptions of support from family, friends, and significant others (e.g., “My friends really try to help me”, “I can talk about my problems with my family”). Participants responded to each item using a 1 (*very strongly disagree*) to 7 (*very strongly agree*) scale. Total scores were calculated as the sum of all items. The scale demonstrated good reliability (ω_*u*_ = .89).

#### Friendship network satisfaction scale

Relationship satisfaction was measured using the Friendship Network Satisfaction Scale (FNSS)^58^. This scale is a 14-item self-report measure of satisfaction with current friendships (e.g., “I feel close to my friends”, “My friends and I go out and do things together”). Participants responded to each item using a 0 (*not at all agree*) to 5 (*completely agree*) scale. Total scores were calculated as the sum of all items. The scale showed good reliability (ω_*u*_ = .88).

### Procedure

All subjects completed informed consent, the robust averaging task, and then the self-report measures assessing PLE, social connection, and demographic characteristics. To ensure data quality and sustained engagement on the self-report surveys, participants were presented with three attention-check items embedded within the questionnaires. There was a technical error with one question, but the other two indicated that inattention was rare with *n* = 5 out of 206 participants missing one of the two attention check questions. The low failure rate indicates that inattention was minimal and unlikely to influence the overall pattern of results.

### Data analysis

To characterize the relationship between self-reported psychotic-like experiences and social connection, we calculated Pearson correlations among the self-report measures (Table 2). This allowed us to understand convergence/divergence between different variable associations in our dataset. To characterize task performance, we quantified each participant’s overall accuracy (proportion of correct responses). We then examined performance as a function of variance (low vs. high) and mean (low vs. high) with a repeated-measures ANOVA. To evaluate whether psychotic-like experiences were associated with task performance, we computed zero-order correlations between overall accuracy and each PLE measure. We further tested whether trial-level variance moderated the relationship between PLEs and performance by fitting random intercept mixed-effects models that included the PLE measure, variance, and their interaction.

**Table 2:** Descriptive statistics and correlations for study measures.

Measure	*n*	*M*	*SD*	Range	1	2	3	4	5	6	7
1. RGPTS-r	206	9.17	7.07	0–30	–						
2. RGPTS-p	204	4.68	6.71	0–36	.78**	–					
3. PDI	206	4.49	3.13	0–14	.41**	.39**	–				
4. CAPS	206	2.27	2.56	0–12	.26**	.25**	.61**	–			
5. ULS	206	36.91	10.04	20–67	.40**	.33**	.31**	.11	–		
6. MSPSS	206	65.96	13.00	34–84	− .25**	− .25**	− .13	− .10	− .67**	–	
7. FNSS	206	53.47	12.59	17–70	− .18**	− .14*	− .12	.05	− .68**	.54**	–

RGPTS-r, Revised Green Paranoid Thoughts Scale-Reference; RGPTS-p, Revised Green Paranoid Thoughts Scale-Persecution; PDI, Peters Delusion Inventory; CAPS, Cardiff Anomalous Perceptions Scale; ULS, UCLA Loneliness Scale; MSPSS, Multidimensional Scale of Perceived Social Support; FNSS, Friendship Network Satisfaction Scale.

*p ≤ .05. **p < .01.

Following Larsen et al.^8^, we determined how each face influenced participants’ decisions on a trial-by-trial basis for each trial type (i.e., trial-wise manipulations of variance, mean, and valence). To do so, we rank ordered the faces for each trial by value so that more extreme faces were either closer to the most extreme negative face (ranks closer to 1) or the most extreme positive face (ranks closer to 8). Next, for each participant we conducted several separate sets of logistic regression models, corresponding to different combinations of our predictors, to estimate how individual face ranks contributed to trial-by-trial decisions. For each combination of predictors (e.g., valence, valence and mean, etc.), trials were divided by task condition combinations and logistic regressions were conducted on each subset of data for each participant. For example, to generate the beta weights for the analysis testing the impact of variance and mean, we conducted four logistic regression models for each participant using trials corresponding to each combination of the conditions: high variance-high mean, high variance-low mean, low variance-high mean, and low variance-low mean. These subsets of data were used in the logistic regression models whereby participants’ trial decisions (i.e., judging the display as more negative or more positive on average) were predicted by the rank-ordered emotional intensity values of the eight faces. This generated eight beta weights per participant per condition combination (e.g., low variance-low mean), representing the relative influence of each face rank on choice for that specific condition combination. Each set of beta weights were used in the corresponding group-level analyses according to which task characteristics were being tested. When testing overall effects (i.e., the quadratic effect between face rank and beta weight), following Larsen et al., we collapsed across the beta weights estimated separately for high and low variance trials. Beta weights were normalized by their root mean square (RMS) to account for individual differences in overall weight magnitude. We did not expect face valence to impact robust averaging and so we report those results in the Supplementary Material. We also note that since we generated the beta weights for a maximum of two task variables at a time to avoid model nonconvergence due to the small number of trials used to estimate weights with three task variables (e.g., high variance, high mean, positive valence trials), we were unable to explore three-way interactions between variance, mean, and valence.

As a way of checking whether the participant-level logistic regressions demonstrated good fit to the data, we performed a likelihood ratio test and Hosmer–Lemeshow Test on the logistic regression models that were used to generate the beta weights for the main analyses (i.e., regressing trial decisions on face rank and variance). The likelihood ratio test revealed that 97% of the individual logistic regressions showed good model fit (*p*s < .05). Similarly, the Hosmer–Lemeshow Test revealed that 96% of the individual logistic regressions were well calibrated to the data (*p*s < .05). Together, these data indicate that for nearly all participants, model fit/calibration was adequate. As a point of comparison, we ran the same two model fit metrics on the Larsen et al. data available on the OSF (https://osf.io/9vp37/overview). We found that 100% of their participants’ individual logistic regressions showed good model fit with the likelihood ratio test, and that 72% of the individual logistics regressions were well calibrated according to the Hosmer–Lemeshow Test.

For analysis, we treated the mean as a categorical variable (low, high), which we determined by taking the absolute difference between the mean 8-face array value and the midpoint value (25) and then performing a median split. Low and high variance sets were defined based on the standard deviation of the individual face emotion intensity values within each 8-face array (*SD* ≈ 5 for low variance; *SD* ≈ 15 for high variance).

If participants use robust averaging, we expect that face ranks near the mean face value (i.e., “inlying” face ranks) would be more heavily weighted during decision-making than face ranks further away from the mean value (i.e., “outlying” face ranks). To evaluate this style of decision-making, following others^8,9^, we used two analytic methods whose findings should converge in the presence of robust averaging. First, we used regression to assess for a quadratic association between face rank and beta weight, such that more extreme faces (e.g., outlying ranks of 1 and 8) received less weight than more inlying ranks. As face rank was a repeated measure, we conducted mixed-effects models including a random intercept for participant. These models indicated that there was essentially no detectable variability across participants beyond the fixed effects. Thus, we proceeded using standard fixed effects models. We repeated this analysis including terms for variance, mean, and their interaction. Significant interactions were probed with simple slopes analysis.

Second, we calculated the mean beta weights for inlying (ranks 3–6) versus outlying (ranks 1, 2, 7, 8) faces. These values were submitted to a paired samples Welch’s *t*-test. To evaluate the impact of variance, we conducted a repeated-measures ANOVA with inlyingness, variance, and their interaction as the predictors and the participant-level logistic regression beta weights as the outcome. To evaluate the impact of variance and mean, we conducted another repeated-measures ANOVA with inlyingness, variance, mean, and the interaction between these terms as the predictors and the participant-level logistic regression beta weights as the outcome.

To evaluate whether robust averaging is associated with PLE or social connection, we used the analytic approaches described above. Specifically, we conducted regression models predicting beta weights from face rank (quadratic term), an individual PLE or social connection measure, and their interaction. We conducted an additional regression model that also included a term for variance and its interaction with other terms in the model (face rank, individual PLE/social connection measure). Using the inlying/outlying analytic strategy, we conducted another set of regressions predicting beta weight by inlyingness, an individual PLE or social connection measure, and their interaction. We similarly conducted an additional regression model that included a term for variance and its interaction with the other terms in the model.

## Results

### Task performance

**Fig. 2:**
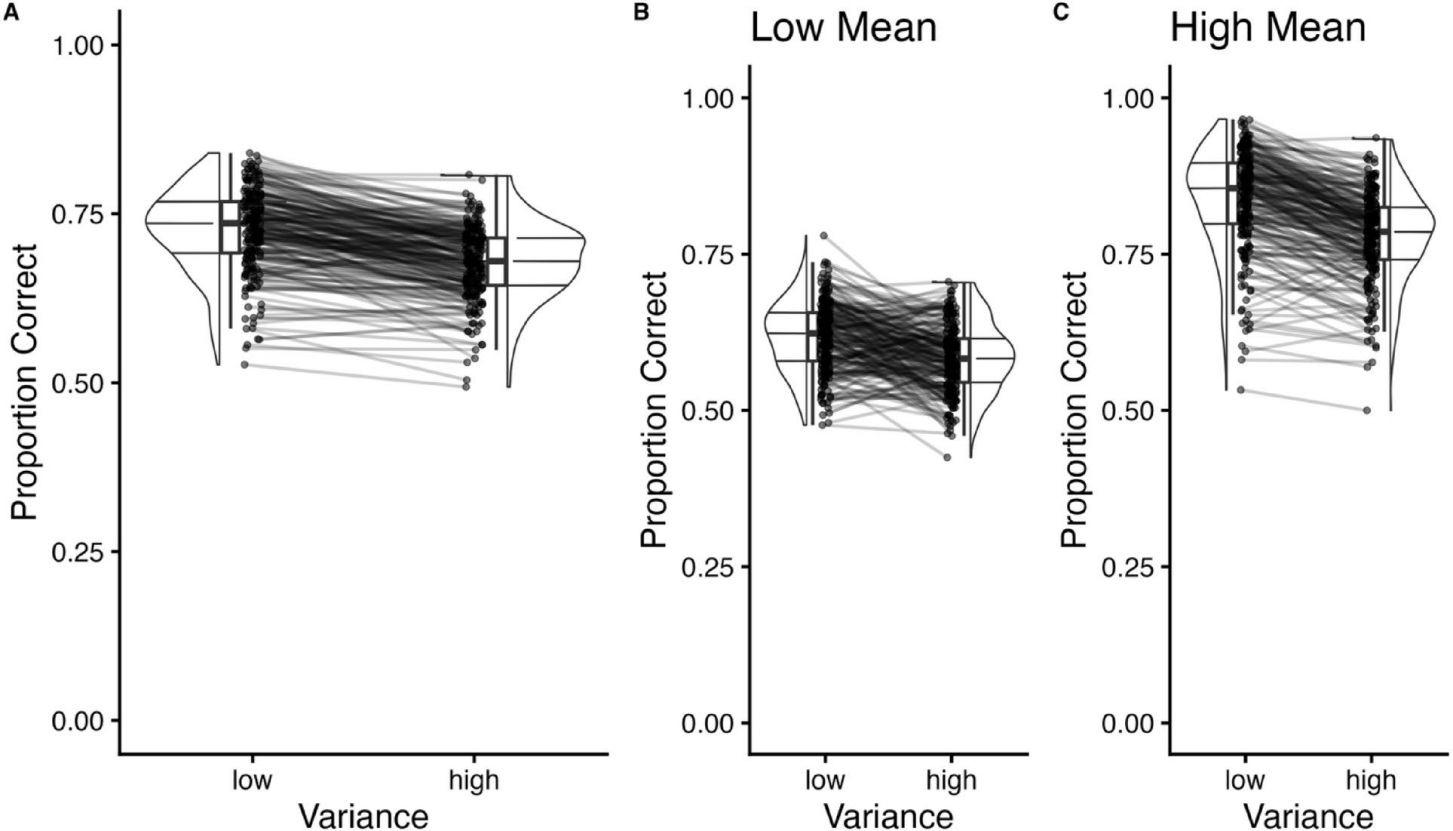
Task accuracy. (**A**) Proportion correct as a function of variance (low, high). Black dots represent individual data points with black lines connecting paired data points from the same participant. (**B**) Proportion correct as a function of variance for low mean trials. (**C**) Proportion correct as a function of variance for high mean trials.

All participants demonstrated good accuracy on the task (*M*_proportion correct_ = .70, *SD* = .46). We tested for differences in accuracy across manipulations of variance and mean. We observed a significant main effect of variance, *F*(1, 205) = 243.59, *p* < .001, $\eta_{{\mathrm{G}}}^{2}$ = 0.11, mean, *F*(1, 205) = 2888.03, *p* < .001, $\eta_{{\mathrm{G}}}^{2}$ = .70, and their interaction, *F*(1, 205) = 22.65, *p* < .001, $\eta_{{\mathrm{G}}}^{2}$ = .01. Post-hoc paired *t*-tests revealed that individuals were more accurate for the low variance trials (*M* = .73, *SD* = .45) compared to the high variance trials (*M* = .68, *SD* = .47), *t*(205) = 15.50, *p* < .001, *d* = 1.08 (Fig. 2A). Individuals were also more accurate for the high mean (greater emotion intensity) trials (*M* = .81, *SD* = .40) compared to the low mean trials (*M* = .60, *SD* = .49), *t*(205) = 53.80, *p* < .001, *d* = 3.75. Regarding the interaction, we found that variance had a greater impact on performance on high mean trials (low variance *M* = .84, *SD* = .37, high variance *M* = .78, *SD* = .42), *t*(205) = 17.40, *p* < .001, *d* = 1.21) versus low mean trials (low variance *M* = .62, *SD* = .49, high variance *M* = .58, *SD* = .49, *t*(205) = 7.92, *p* < .001, *d* = 0.55; Fig. 2B, C).

We examined whether performance differed as a function of PLE and found that accuracy was positively correlated with CAPS, *r* = .21, *p* = .003, but not the other PLE variables (*p*s > .057). This finding is consistent with some work demonstrating increased perceptual sensitivity in psychosis-risk conditions^59–61^. We tested whether this association was impacted by trial variance using a mixed-effect model, but did not find a PLE by variance interaction (*b* = 0.00001, *t* = 0.01, *p* = .996).

### Robust averaging: quadratic association between face rank and beta weight

**Fig. 3:**
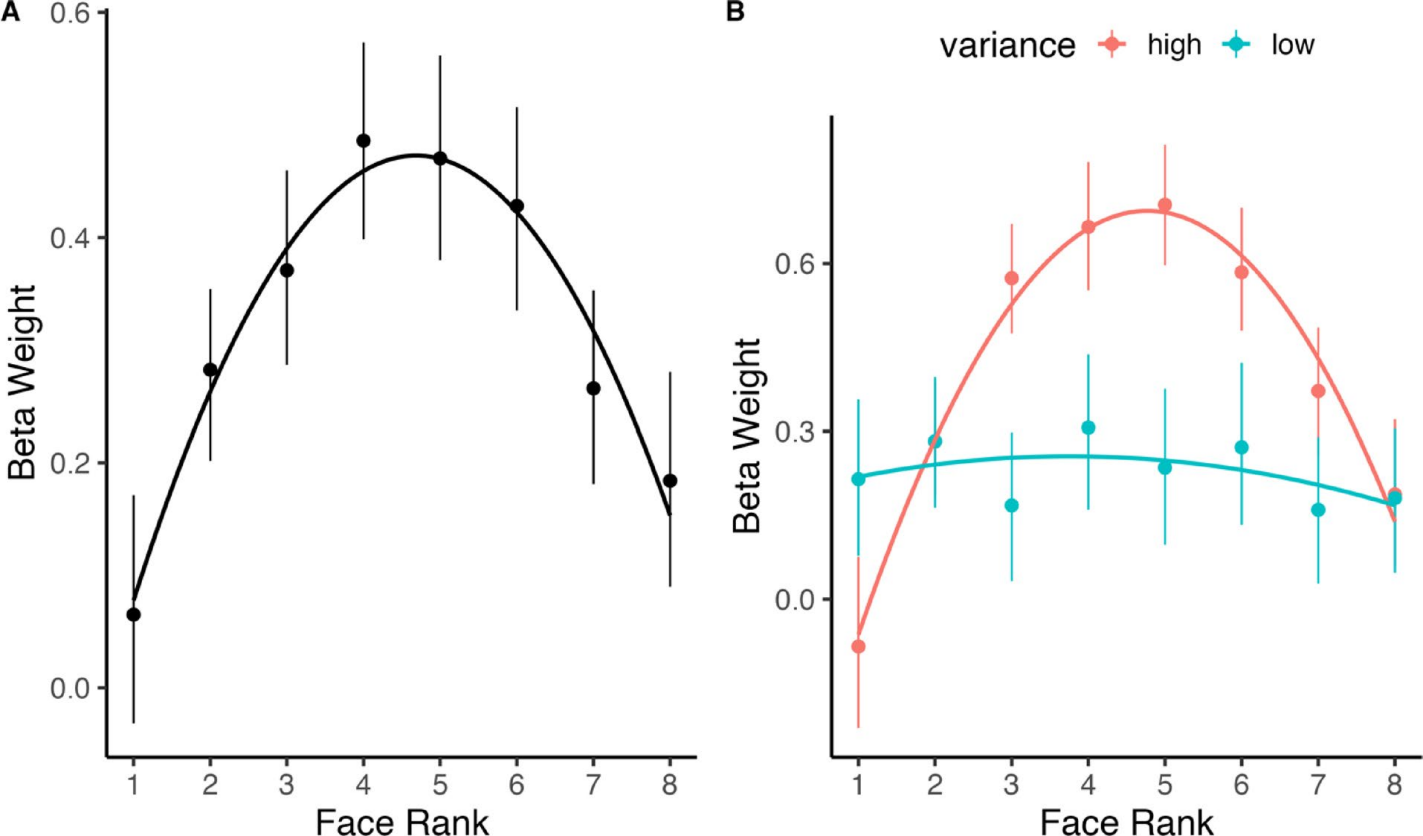
Quadratic effect of face rank on beta weight. (**A**) The impact of face rank on beta weight. The black line depicts the quadratic regression line and datapoints depict the mean ± 95% CI. The association is quadratic in nature whereby face ranks closer to the mean are weighted more heavily during decision-making, while face ranks further from the mean are weighted less heavily during decision-making, which can be taken as evidence of robust averaging. (**B**) The impact of face rank on beta weight as a function of variance (blue = low variance, red = high variance). Robust averaging is observed for high, but not low variance trials.

We examined the effect of face rank on beta weights using a linear regression with beta weight as the outcome and a quadratic term for face rank as the predictor. The quadratic term (inverted u-shape) was significant, *b* =  − 7.57, *SE* = .94, *t* =  − 8.06, *p* < .001 (Fig. 3A), indicating downweighting of outlying element ranks that defines the presence of robust averaging.

We then examined whether the quadratic effect between element number and beta weight differed by variance (Fig. 3B). We found a significant interaction between the quadratic term and variance, *b* = 12.50, *SE* = 1.86, *t* = 6.74, *p* < .001, which we probed by conducting follow-up regressions separately by variance. In the high-variance condition there was a significant quadratic effect, *b* =  − 9.77, *SE* = .88, *t* = 11.10, *p* < .001. In the low variance condition, there was not a significant quadratic effect, *b* =  − .93, *SE* = .98, *t* = .96, *p* = .340. These results indicate robust averaging occurs for high variance conditions but not low variance conditions. In other words, outlying elements are adaptively downweighted, but only when the reliability of the array is low. We found no three-way interaction between element rank, variance, and mean on beta weight, *b* =  − .03, *SE* = 3.86, *t* = .01, *p* = .994, indicating that the interaction between element rank and variance does not differ across levels of the mean.

### Robust averaging: effect of inlying versus outlying face ranks

**Fig. 4:**
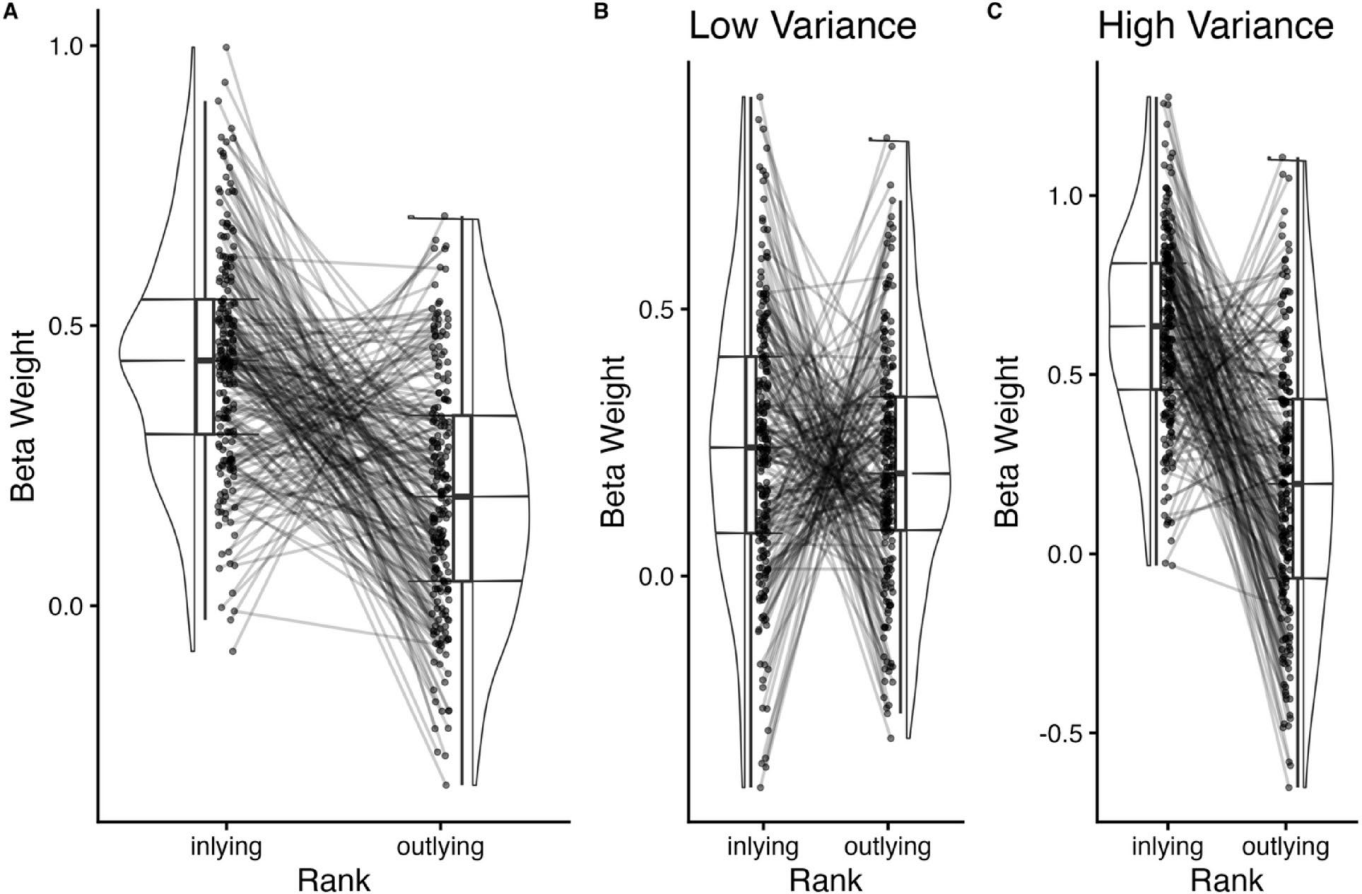
Beta weight as a function of inlying versus outlying face rank. (**A**) Beta weight as a function of inlying (face ranks 3–6) versus outlying (face ranks 1, 2, 7, 8) face ranks. Black dots represent individual data points with black lines connecting paired data points from the same participant. Higher beta weights for inlying versus outlying face ranks can be taken as evidence of robust averaging. (**B**) Beta weight as a function of inlying versus outlying face rank for low variance trials. (**C**) Beta weight as a function of inlying versus outlying face rank for high variance trials.

Next, we tested for the presence of robust averaging by comparing beta weights for inlying (face ranks 3–6) versus outlying (face ranks 1, 2, 7, 8) faces. Consistent with the findings of the regression analyses above, a paired-samples *t*-test revealed that beta weights were significantly higher for inlying elements (*M* = .44, *SD* = .20) than outlying elements (*M* = .20, *SD* = .21), *t*(205) = 9.99, *p* =  < .001, *d* = .70 (Fig. 4A). We then examined whether the effect of inlying/outlying rank on beta weight differed by variance using a 2 inlying/outlying × 2 variance (low, high) repeated measures ANOVA (Fig. 4B, C). We observed a significant main effect of inlyingness, *F*(1, 205) = 99.76, *p* < .001, $\eta_{{\mathrm{G}}}^{2}$ = .16, variance, *F*(1, 205) = 196.80, *p* < .001, $\eta_{{\mathrm{G}}}^{2}$ = .10, and their interaction, *F*(1, 205) = 78.94, *p* < .001, $\eta_{{\mathrm{G}}}^{2}$ = .12. Post-hoc paired *t*-tests revealed that for high variance trials, beta weights were significantly higher for inlying elements compared to outlying elements, *t*(205) = 12.20, *p* < .001, *d* = .85. For low variance trials, there was not a significant difference in beta weights between inlying and outlying elements, *t*(205) = 1.31, *p* = .191, *d* = .09. Similar to the quadratic regression findings, these results suggest that robust averaging occurs only when stimuli are more variable.

Next, we evaluated the impact of inlyingness, variance, and mean on trial-by-trial decisions. In this model, unlike the effects of inlyingness, *F*(1, 205) = 66.32, *p* < .001, $\eta_{{\mathrm{G}}}^{2}$ = .06, and variance, *F*(1, 205) = 167.89, *p* < .001, $\eta_{{\mathrm{G}}}^{2}$ = .05, the mean of the array did not impact trial-by-trial decisions, *F*(1, 205) = 3.19, *p* = .076, $\eta_{{\mathrm{G}}}^{2}$ = .001. The mean did however moderate the effect of inlyingness, *F*_inylingness*mean_(1, 205) = 5.08, *p* = .025, $\eta_{{\mathrm{G}}}^{2}$ = .005. Although beta weights were higher for inlying versus outlying faces across both levels of the mean, the effect was stronger in the low mean trials, *t*(411) = 8.01, *p* < .001, *d* = .39, compared to the high mean trials, *t*(411) = 4.11, *p* < .001, *d* = .20. This pattern of results could suggest that when emotional intensity is low overall (i.e., a mix of neutral and slightly valenced faces), inlying elements may provide more informative cues, leading to participants weight those elements more strongly. Conversely, when emotional intensity is overall high (i.e., a mix of more intensely valenced positive and negative faces), the difference between inlying and outlying faces may be more salient in a way that does not necessitate as much reliance on inlying elements during decision-making. We did not observe a three-way interaction between inlyingness, variance, and mean, *F*_inylingness*variance*mean_(1, 205) =  .68, *p* = .411, $\eta_{{\mathrm{G}}}^{2}$ = .0005.

### Robust averaging and PLE

To examine the association between psychotic-like experiences and robust averaging, we used the same strategy as above, testing the interaction of individual PLE measures with the quadratic effect of face rank in one analysis, and the interaction of individual PLE measures with inlyingness in another analysis. We conducted these models separately for each of the PLE measures. We found no associations between any measure of PLE and robust averaging in the quadratic regression or inlying/outlying rank analysis (*b*s =  − .04 to .01, *p*s > .06), nor an effect of PLE when including variance in the models (*b*s =  − .21 to .33, *p*s > .20; Supplementary Material). To rule out the possibility that individuals higher in PLE demonstrate altered robust averaging that they learn to adjust over time with feedback from the task, we evaluated whether PLE impacted the extent of robust averaging for early versus late trials (i.e., trials 1–250 versus trials 251–500; Supplementary Material). Neither analytic approach revealed a time by PLE interaction indicating that PLE did not impact learning over the course of the task.

### Robust averaging and social connection

To examine the association between social connection and robust averaging, we repeated the analyses above substituting an individual PLE measure for an individual social connection measure. We conducted these models separately for each of the social connection measures. There were no associations between any measure of social connection and robust averaging in the quadratic regression or inlying/outlying rank analysis (*b*s =  − .03 to .01, *p*s > .20), nor an effect of social connection when including variance in the models (*b*s =  − .08 to .14, *p*s > .20; Supplementary Material).

## Discussion

In our everyday lives, we are bombarded with social information. Sometimes this information is readily interpretable, allowing us to select social behaviors that are clearly warranted by the social situation. Oftentimes though, social information is noisy and inscrutable, creating a predicament for choosing appropriate social behaviors. One perceptual mechanism that may help us solve this challenge is robust averaging, an analytic feature of perception where we downweight outlying or extreme information when generating ensemble summaries. Not everyone might use this mechanism in the same way—it has been proposed that individuals with psychotic experiences and disorders are “bad statisticians,” accepting weak or noisy evidence as valid due to lowered decision thresholds. In consideration of these ideas, here, the current study examined whether robust averaging occurs for social information, is associated with psychotic-like experiences, and impacts social connection. We used a facial averaging task in which the strength and reliability changed to assess how individuals integrate information when making decisions about social information.

Consistent with previous work^8,9^, we found that individuals demonstrated robust averaging when forming ensemble summaries to make decisions about social information. Specifically, across two complementary analytic strategies, participants downweighted outlying faces further from the mean of the array and upweighted inlying faces closer to the mean of the array. Individuals may utilize robust averaging in processing social stimuli because it allows information to be conveyed rapidly without relying on consciously representing all individual components of a scene^14,62–64^ and without losing the specificity and detail expected from the limits of visual short-term memory and attention^65^. This can be socially adaptive in any situation that requires “reading a room”, such as detecting changes in the collective mood of a group during conversation about a sensitive topic, adapting communication to a group of potential employees during an interview, reading cues from the crowd while giving a talk, or those that might have implications for one’s safety, such as being accosted by a group of individuals at night and needing to rapidly infer their intention.

Not all scenarios may benefit from robust averaging though. In situations where social information is consistent and clear, downweighting particular pieces of social information may be unnecessary, inefficient, and unhelpful. In line with this idea, we observed robust averaging only for high variance trials, where the consistency of the faces in terms of emotion were low, but not low variance trials, where any single face provided similar information as others. This is consistent with previous research indicating the presence of robust averaging only for high-variance conditions^8,9^. These findings are also consistent with statistical perspectives that state when sensory signals are noisy and variable, reducing the signal of the outlying evidence during information integration protects decision making from being vulnerable to irrelevant information^66^. Conversely, when sensory signals are more uniform, extreme values and elements may be treated more similarly to the true signal due to less irrelevant information being conveyed. Put in other terms, in situations with high stimulus similarity and redundancy, it may be a waste of resources to average perceptual information.

Regarding the potential alteration of social robust averaging in the psychosis spectrum, unlike Larsen et al.^8^, we did not find that robust averaging was associated with PLE. This suggests that while PLE affects one’s ability to extract summary information for low-level perceptual features, PLE does not appear to impact one’s ability to form similar statistical summaries for social information. Although this may seem contrary to other previous work suggesting a generalized evidence integration alteration in psychosis characterized by the tendency to attribute increased meaning to weakly supported evidence^26,67–70^, we see at least two explanations for these findings, one related to our sample and the other to our task’s stimuli. Regarding our sample, it is possible that PLE impacts social robust averaging, but only at extremely high levels of PLE. Only a small number of our participants met established clinical cutoffs for the RGPTS and PDI, and few participants met the cutoff for the high hallucination-proneness group used in Larsen et al., precluding us from evaluating differences between participants with and without clinically significant levels of PLE with a reasonable amount of statistical power. Regarding task stimuli, faces are in many ways a unique stimulus in how they compel our attention, in our preference for them, in the inordinate amount of time we spend looking at them, and in our relative expertise in recognizing them^71–73^. The degree of our exposure to faces and our experience individuating them and the affect they express may compensate for any subtle PLE-related alterations in how facial affect information is integrated, masking what could be small differences in social robust averaging that we were underpowered to detect. It is also worth noting that individuals with schizophrenia exhibit color perception deficits^74^, including increased errors in discriminating between colors or delays in color recognition, which could have contributed to Larsen et al.’s findings of reduced robust averaging for color information in hallucination-prone individuals.

Despite the intuitive utility of robust averaging for adaptive social behavior, we did not find that robust averaging was associated with any social connection measures. Because we measured social connection at the broadest level, other unmeasured processes likely come to bear, which may have masked potential associations. Further, the association between aspects of social perception, like robust averaging, and social functioning, like loneliness and perceived social support, is not necessarily a direct link. Although altered ensemble perception of social information may lead individuals to draw faulty conclusions about the emotion, mental state, or intentions of a group, it need not contribute to maladaptive social behavior. For distorted summary representations to impair social functioning, an individual might need to make consistent and pervasive attributional errors, act reflexively or impulsively in social situations, and/or exhibit overconfidence in their social judgments, failing to consider the inherently fuzzy nature of social information. As such, individuals who generate only the occasional faulty summary perception of social information or does so consistently, but not to a markedly altered degree may not experience a social functioning impairment. Likewise, cognitive control – the class of mechanisms that organize and guide thought and behavior in accordance with one’s goals^75–77^—may impact robust averaging whereby those with high levels of cognitive control who do not act impulsively in social situations and who flexibly maintain multiple interpretations of social information may be able to compensate for any disrupted robust averaging process. The same may be true for individuals who excel in other social cognitive processes (e.g., mentalizing) that can inform and correct initial social interpretations created by altered robust averaging. Additionally, social functioning was measured through self-report measures only; it is possible that social functioning as rated by others or objective tests and measures of social cognition may show different associations with robust averaging.

We note two final considerations that apply to the lack of observed associations between robust averaging, social connection, and PLE. First, although there may be a wealth of social contexts in which robust averaging is appropriate, adaptive, or necessary, there too are social situations in which outlying signals are most informative and responding according to a group average may be maladaptive. For example, if one’s goal was to identify a suspicious person in a crowd or help someone who was clearly lost in a group of people who were generally on the same page, upweighting outlying information in these contexts would be helpful. What may characterize successful social behavior and what may be the primary alteration in the psychosis spectrum is the flexible switching between these modes of perception—downweighting outliers and making decisions based on the statistical mean versus upweighting outliers and making decisions based on what is irregular—depending on the context.

Second, stimuli in this study were presented in a circle and thus enhanced perceptual organization of the faces, which may have made it easier to average them. In contrast, in the real-world, stimuli are rarely organized and processed in this way^27,78,79^. Additionally, schizophrenia is reliably associated with perceptual organization impairments, but not when the structure of the stimulus is symmetrical or a platonic form, such as a perfect circle or square^80^. We speculate that if the stimuli were not presented in a perfect circle, which would have required participants to create a scan path through the stimuli and utilize working memory and executive control, differences might have emerged between individuals with PLE and those without.

## Future directions

As we previously noted, only a small number of our participants met established clinical cutoffs for the PLE measures, and this may have contributed to the lack of an association between robust averaging and PLE. It may be that robust averaging is altered only at extremely high levels of PLE, such as those found in psychotic spectrum disorders. As such, it would be valuable for future studies of robust averaging to look at social stimuli in a schizophrenia spectrum disorder sample, or at least one with a higher number of participants meeting PLE clinical cutoffs.

Additionally, social disconnection in psychotic spectrum disorders may more accurately reflect challenges with flexibly switching between the adaptively downweighting and adaptively upweighting social information depending on the context. Future research should examine the ability of individuals to appropriately and efficiently switch between theses modes when judging social information and test its association with social connection and PLE.

Beyond how different social contexts may determine the appropriateness of robust averaging, there may also be certain features of the social target that trigger or suppress robust averaging, such as the number of targets a person is making inferences about, the perceiver’s similarity to or familiarity with the targets, and the emotional lability of the targets, among other features. It would be interesting for future work to explore the range of features relating to social targets that affect the robust averaging process.

While the present study aimed to build on existing literature to identify whether robust averaging occurs for social information, there are many additional factors to consider. While our study focused on valenced social stimuli in an effort to replicate the forms of facial expressions routinely experienced in day-to-day life, judgements about neutral faces and other social versus non-social stimuli could provide valuable information about the mechanisms of (non-valenced) social and non-social perceptual integration when compared to valenced social stimuli. Additionally, the faces we come across in our day-to-day lives are dynamic and may express a combination of emotions. Therefore, future work examining the extent to which robust averaging occurs for more ecologically valid, dynamic faces would provide valuable insight into how robust averaging may impact day-to-day life.

Future research would also benefit from including a range of other measures to clarify variables that may either contribute to or result from social robust averaging. For example, a limitation of the present study is that we did not include direct measures of anxiety or mood, which may bias judgments of crowd emotion. Previous research suggests that socially anxious individuals tend to rate facial crowds as more negative compared to controls^81,82^. However, research suggests that social anxiety does not appear to affect precision when extracting ensemble-level emotional information (i.e., distinguishing objectively negative crowds from objectively positive crowds)^82^. Nonetheless, future research should include measures of anxiety and mood to clarify if robust averaging of higher-order stimuli (e.g., faces) is impacted by individual differences (i.e., anxiety). Additionally, while the present study aimed to identify whether perceptual averaging differences vary according to psychotic-like experiences, probability-based reasoning tasks (e.g., “jumping-to-conclusions” paradigm) tap related integrative decision-making at a higher cognitive level. Future research should explore whether variability in perceptual averaging corresponds with probabilistic reasoning biases seen in schizophrenia-spectrum disorders to determine whether there is a common evidence-weighting and integration alteration occurring under uncertainty. Future studies could incorporate explicit executive or attention-control measures (e.g., working memory, task-switching) to further assess how these cognitive abilities relate to robust averaging performance. Such work would help clarify the contributions of higher-order cognitive control to social perceptual processes and individual differences in performance.

Finally, although our findings provide insight into social information processing in a young, primarily Asian and White student sample with low levels of loneliness and PLEs, caution is warranted in generalizing to other populations. Future work should examine whether these patterns replicate in more ethnoracially and educationally diverse samples with greater variability in social connection and mental health.

## Conclusion

Here, we find evidence of robust averaging during a facial affect averaging task, specifically under conditions of high, but not low, stimulus variability, suggesting that this feature of perception extends to social information processing. This effect was not associated with general measures of social connection nor different domains of PLE. It would be useful for future work to investigate robust averaging for externally valid, dynamic social information, robust averaging’s ability to explain individual differences in a range of social functions like speed of social decision-making and one’s ability to “read a room,” robust averaging’s alteration in individuals with psychotic disorders, and whether flexibly switching between modes of downweighting versus upweighting statistically outlying information based on social context is associated with social connection and PLE.

## Supplementary Information

Below is the link to the electronic supplementary material.

Supplementary Material 1

## Data Availability

The datasets generated and analyzed during the current study are available in the Open Science Framework, https://osf.io/w596j/.
