# Supplementary material for: Robust averaging of emotional faces and its association with psychotic-like experiences and social connection
Source: Sci Rep. 2026 Jan 10;16:4965. doi: 10.1038/s41598-026-35374-z (PMC12877069; doi:10.1038/s41598-026-35374-z)
Supplement: Supplementary file 1 — Supplementary Material 1 [file 41598_2026_35374_MOESM1_ESM.docx]

**Supplemental Material**

**Robust Averaging of Emotional Faces and its Association with Psychotic-Like Experiences and Social Connection**

**The Effect of Valence**

We tested the effects of element rank and valence on beta weight using a linear regression (Supplemental Fig. 1). There was not a significant main effect of valence, *b* = 0.02, *SE* = 0.02, *t* = 0.93, *p* = 0.35), or a significant interaction between the quadratic term and valence (β = 0.44, *SE* = 1.98, *t* = 0.22, *p* = 0.83). These results suggest that robust averaging does not differ by valence. Follow up simple slopes analyses revealed that for negative valence trials, beta weights increased with element rank (β = -4.18, *p* < .001), while for positive valence trials, beta weights decreased with element rank (β = -3.87, *p* < .001). These results suggest that for negative valence trials, individuals tend to weight the more positive faces higher, and for the positive valence trials individuals tend to weight the more negative face higher. Thus, if most of the faces are of one valence, individuals adjust their weightings based on the faces of a different valence. There was not a significant three-way interaction when variance was added to the model, β = -0.48, *p* = .90, indicating that the effect of element rank on variance does not differ across valence. We also conducted a 2x2 repeated-measures ANOVA to examine the effects of inlying/outlying rank and valence on beta-weights (Supplemental Fig. 2). There was not a significant main effect of valence, *F*(1, 205) = 0.274, *p* = .601, *ges* = .0002, or a significant interaction between inlying/outlying rank and valence, *F*(1, 205) = 0.19, *p* = .662, *ges* = .0003. These results suggest that robust averaging does not differ by valence.

**Supplemental Figure 1**

*Quadratic effect of beta weight by face rank across valence conditions*

**Supplemental Figure 2**

*Beta weight by inlying versus outlying ranks across valence conditions*


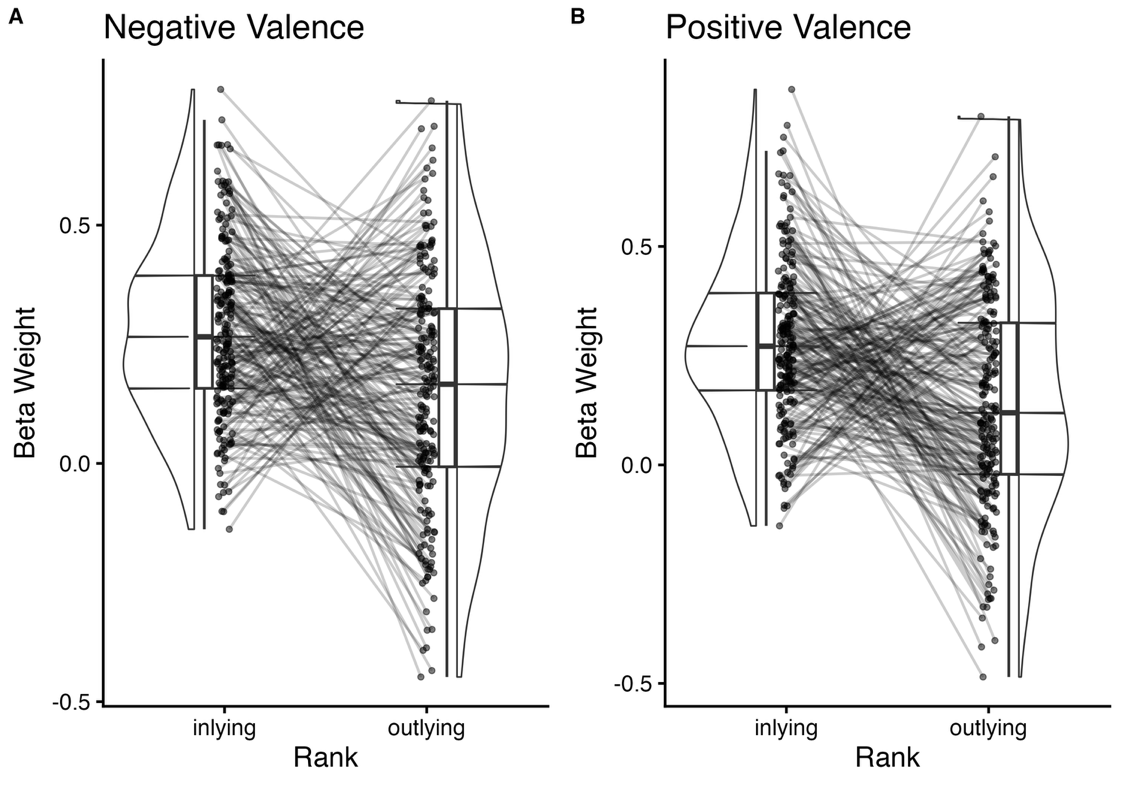


**Learning Effects**

To rule out the possibility of group differences being masked by learning effects, we looked at robust averaging as a function of learning (early vs. late task trials). There was not a significant main effect of time, *F*(1, 205) = 0.227, *p* = .63, *ges* = .0001, or a significant interaction between inlying/outlying rank and time, *F*(1, 205) = 0.38, *p* = .54, *ges* = .0003, variance and time, *F*(1, 205) = 1.43, *p* = .23, *ges* = .0004, or their three-way interaction, *F*(1, 205) = 1.09, *p* = .30, *ges* = .001. These results suggest that there was no differential impact of learning on decision-making (Supplemental Fig. 3). We found no effect of PLE when including time in the models (*b*s=-.12-.20, *p*s>.65; Supplemental Table 1).

**Supplemental Figure 3**

*Beta weight by inlying versus outlying ranks across variance and time conditions*


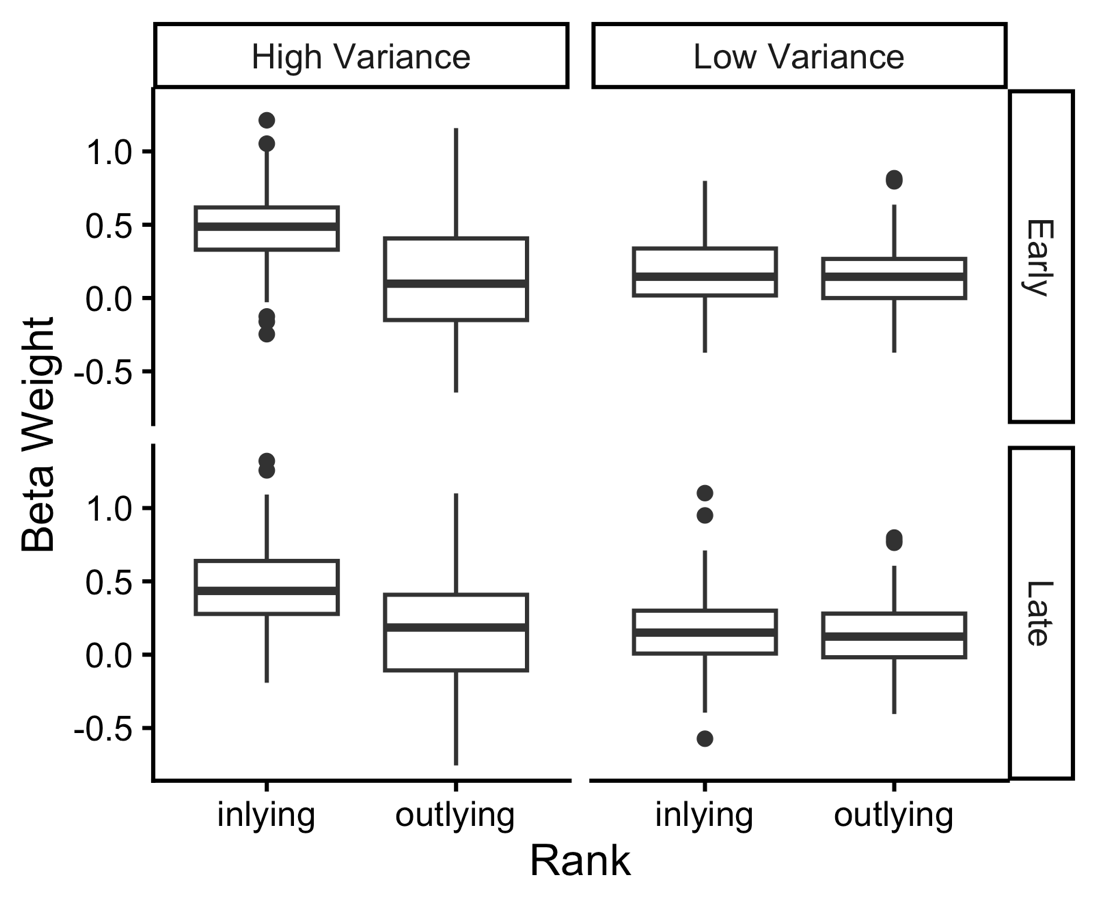


**Supplemental Table 1**

*Psychotic-like experiences and robust averaging results across time (early vs. late trials)*

| PLE Measure | Estimate (*b*) | *SE* | *t* | *p* |
| --- | --- | --- | --- | --- |
|  | | | | |
| *Quadratic Regression* | | | | |
| RGPTS-Reference x Face Rank Quadratic Effect x Time | -.12 | .27 | -.45 | .651 |
| RGPTS-Persecution x Face Rank Quadratic Effect x Time | -.03 | .29 | -.11 | .910 |
| PDI x Face Rank Quadratic Effect x Time | .20 | .62 | .33 | .741 |
| CAPS x Face Rank Quadratic Effect x Time | .15 | .76 | .20 | .844 |

**Supplemental Table 2**

*Psychotic-like experiences and robust averaging results*

| PLE Measure | Estimate (*b*) | *SE* | *t* | *p* |
| --- | --- | --- | --- | --- |
|  | | | | |
| *Quadratic Regression* | | | | |
| RGPTS-Reference x Face Rank Quadratic Effect | -.12 | .13 | -.89 | .373 |
| RGPTS-Reference x Face Rank Quadratic Effect x Variance | .26 | .26 | .99 | .319 |
| RGPTS-Persecution x Face Rank Quadratic Effect | -.07 | .14 | -.52 | .602 |
| RGPTS-Persecution x Face Rank Quadratic Effect x Variance | .22 | .28 | .79 | .432 |
| PDI x Face Rank Quadratic Effect | -.40 | .30 | -1.34 | .182 |
| PDI x Face Rank Quadratic Effect x Variance | .33 | .59 | .56 | .578 |
| CAPS x Face Rank Quadratic Effect | -.03 | .37 | -.09 | .925 |
| CAPS x Face Rank Quadratic Effect x Variance | -.21 | .73 | -.29 | .774 |
|  | | | | |
| Inlying/Outlying Ranks | | | | |
| RGPTS-Reference x Inlying/Outlying | -.003 | .003 | -1.09 | .277 |
| RGPTS-Reference x Inlying/Outlying x Variance | .001 | .005 | .10 | .918 |
| RGPTS-Persecution x Inlying/Outlying | -.004 | .003 | -1.22 | .223 |
| RGPTS-Persecution x Inlying/Outlying x Variance | .00002 | .006 | .004 | .997 |
| PDI x Inlying/Outlying | -.01 | .006 | -1.87 | .062 |
| PDI x Inlying/Outlying x Variance | -.007 | .01 | -.60 | .552 |
| CAPS x Inlying/Outlying | -.004 | .008 | -.54 | .591 |
| CAPS x Inlying/Outlying x Variance | -.02 | .02 | -1.29 | .197 |

**Supplemental Table 3**

*Social connection and robust averaging results*

| Social Connection Measure | Estimate (*b*) | *SE* | *t* | *p* |
| --- | --- | --- | --- | --- |
|  | | | | |
| *Quadratic Regression* | | | | |
| Loneliness x Face Rank Quadratic Effect | -.02 | .10 | -.23 | .819 |
| Loneliness x Face Rank Quadratic Effect x Variance | .14 | .19 | .74 | .463 |
| MSPSS x Face Rank Quadratic Effect | .02 | .07 | .25 | .802 |
| MSPSS x Face Rank Quadratic Effect x Variance | -.03 | .14 | -.20 | .843 |
| FNSS x Face Rank Quadratic Effect | -.03 | .07 | -.40 | .687 |
| FNSS x Face Rank Quadratic Effect x Variance | -.08 | .15 | -.51 | .610 |
|  | | | | |
| Inlying/Outlying Ranks | | | | |
| Loneliness x Inlying/Outlying | .0001 | .002 | .31 | .754 |
| Loneliness x Inlying/Outlying x Variance | -.002 | .004 | -.43 | .666 |
| MSPSS x Inlying/Outlying | -.0005 | .002 | -.29 | .769 |
| MSPSS x Inlying/Outlying x Variance | . 003 | .003 | 1.17 | .244 |
| FNSS x Inlying/Outlying | -.001 | .002 | -.60 | .547 |
| FNSS x Inlying/Outlying x Variance | .002 | .003 | .67 | .506 |
